# Rare cases of primary central nervous system anaplastic variant of diffuse large B-cell lymphoma

**DOI:** 10.1186/s13000-019-0826-0

**Published:** 2019-05-20

**Authors:** Tianqi Xu, Qingge Jia, Yingmei Wang, Yixiong Liu, Donghui Han, Peifeng Li, Jing Ma, Linni Fan, Qingguo Yan, Shuangping Guo, Mingyang Li, Zhe Wang

**Affiliations:** 1 Department of Hematology, Xijing Hospital, Fourth Military Medical University, Xi’an, 710032 China; 2 Second Retired Cadres Sanitarium of Xi’an, Shaanxi Province Military Region, Xi’an, 710032 China; 3 State Key Laboratory of Cancer Biology, Department of Pathology, Xijing Hospital and School of Basic Medicine, Fourth Military Medical University, Xi’an, 710032 China; 4 Department of Urology, Xijing Hospital, Fourth Military Medical University, Xi’an, 710032 China

**Keywords:** Primary central nervous system diffuse large B-cell lymphoma, Anaplastic variant of diffuse large B-cell lymphoma, Concurrent *MYC* and *BCL2* and/or *BCL6* abnormalities, *MYD88* L265P mutation, Poor prognosis

## Abstract

**Background:**

Primary central nervous system (CNS) diffuse large B-cell lymphoma (DLBCL) is a rare intracranial tumor, defined as DLBCL arising from the brain, spinal cord, leptomeninges and eye, with an overall annual incidence of 5 cases per million. The primary CNS anaplastic variant of DLBCL (A-DLBCL) is even less common; to our knowledge, there are only two other case reports in the literature. The aim of this report is to present rare cases of primary CNS A-DLBCL and study their clinicopathologic and genetic features.

**Case presentation:**

We report 3 patients, two men and one woman, aged 54, 55 and 67 years old, with primary CNS A-DLBCL. All 3 patients had a high International Extranodal Lymphoma Study Group (IELSG) score; although the patients were treated with methotrexate-based regimens and/or with radiation therapy, the overall survival was only 2, 5, and 8 months. All 3 patients presented with characteristic features of perivascular space infiltration with bizarre-shaped tumor cells, leading to the diagnosis of primary CNS A-DLBCL. Concurrent of *MYC* and *BCL2* and/or *BCL6* abnormalities and MYC/BCL2 double-expressor DLBCL occurred in all 3 patients; two patients had *MYC*/*BCL2*/*BCL6* triple extra copies, and one patient had *MYC* extra copy and *BCL6* translocation. All 3 patients displayed mutations in *MYD88* L265P and nuclear positivity for RELA, RELB and/or c-Rel, indicating constitutive activation of the NF-κB pathway.

**Conclusions:**

These cases shed light on the unique genetic alterations and biological features of primary CNS A-DLBCL. Patients with primary CNS A-DLBCL may often have a MYC/BCL2 double-expressor and concurrent *MYC* and *BCL2* and/or *BCL6* genetic abnormalities, as well as constitutive activation of the NF-κB pathway. Primary CNS A-DLBCL follows a very aggressive disease course and poor prognosis. In the future, a large number of cases should be analyzed, and the evaluation of molecular genetic characteristics could help with practical and therapeutic implications for primary CNS A-DLBCL.

## Background

Diffuse large B-cell lymphoma (DLBCL) is the most common lymphoma subtype and accounts for 30–40% of adult non-Hodgkin lymphoma (NHL) [1]. Primary central nervous system (CNS) DLBCL is defined as DLBCL arising from the brain, spinal cord, leptomeninges and eye. Primary CNS DLBCL is a rare subtype of B-cell lymphoma, which represents less than 1% of all NHL and 2.4–3% of all brain tumors [2], and is classified as a distinct entity in the WHO classification of tumors in hematopoietic and lymphoid tissues [1]. Statistics shows a peak incidence of patients aged 50 to 60 years (median 56 years old); however, in the recent two decades, there is an increasing incidence of reported patients over the age of 60 years old with primary CNS DLBCL [3]. As it is difficult to make an early diagnosis and because targeted therapy is lacking, primary CNS DLBCL usually shows poorer prognosis than extraneural systemic DLBCL [3].

DLBCL, not otherwise specified (DLBCL, NOS) is morphologically diverse and is generally divided into 3 morphological variants: centroblastic, immunoblastic, and anaplastic [1]. The anaplastic variant of DLBCL (A-DLBCL) is an uncommon morphologic variant that represents approximately 3.4% of all DLBCL cases [4, 5] and is characterized by large to very large polygonal cells with bizarre pleomorphic nuclei that resemble tumor cells of anaplastic large cell lymphoma (ALCL) [1]. Recently, we reported that A-DLBCL had a high frequency of *TP53* mutations and concurrent *MYC* and *BCL2* and/or *BCL6* genetic abnormalities, indicating that the clinicopathologic features and aggressive behavior of A-DLBCL are distinct from the common DLBCL [6].

Because of the rarity, to our knowledge, there are only two case reports describing primary CNS A-DLBCL in the literature [7, 8]. In this study, we reported on 3 patients with primary CNS A-DLBCL and studied their clinicopathologic and genetic features to provide further information for diagnostic and prognostic assessments as well as treatments of this distinctive type of DLBCL.

## Case presentation

### Clinical findings

#### Case 1

A 67-year-old man was admitted to our hospital with intermittent headache for 10 days and hypomnesis for a week. The patient had no B symptoms but was generally in poor condition (Eastern Cooperative Oncology Group (ECOG) performance status =2). Neuroimaging revealed a homogeneously enhancing mass with peripheral signal hyperintensity on the right temporal. Serum lactate dehydrogenase (LDH) level (630 U/L, reference range: 135–215 U/L) and cerebrospinal fluid (CSF) protein concentration (954 mg/L, reference range: 150–450 mg/L) were elevated in the patient. Involvement of deep structures of the brain was not found. The International Extranodal Lymphoma Study Group (IELSG) score [9] was 4 and belonged to the high-risk group. This patient received high-dose methotrexate (HD-MTX) (3.5 g/m^2^) and the concomitant chemotherapy drug idarubicin after surgery. The patient achieved a partial remission according to the response criteria [10] after therapy but died 5 months after the onset of disease.

#### Case 2

A 54-year-old man was admitted to our hospital with a history of right limb weakness for 1 year. The patient had no B symptoms, and the general condition was good (ECOG performance status =0). Neuroimaging showed a noncalcified homogeneously enhancing mass with peripheral signal hyperintensity around the ventricles with associated edema and multiple damaged parts. Serum LDH level (375 U/L) and CSF protein concentration (625 mg/L) were elevated. Involvement of deep structures of the brain was found, and the IELSG score was 4 and belonged to the high-risk group. This patient received HD-MTX (3.5 g/m^2^) and the concomitant chemotherapy drug cytarabine after surgery followed by consolidative whole-brain radiotherapy (40 Gy). He achieved a PR after therapy but died 8 months after diagnosis.

#### Case 3

A 55-year-old woman was admitted to our hospital with history of dizzy and headache for 1 year. The patient had no B symptoms and her general condition was poor (ECOG performance status =3). Neuroimaging revealed a homogeneously enhancing mass with peripheral signal hyperintensity on the interventricular septum and the corpus callosum with associated obstructive hydrocephalus. Serum LDH level (780 U/L) and CSF protein concentration (863 mg/L) were elevated. Involvement of deep structures of the brain was found, and the IELSG score was 4 and belonged to the high-risk group. This patient received HD-MTX (3.5 g/m^2^) and the concomitant chemotherapy drug idarubicin after surgery. She had progressive disease after therapy and died within 2 months of diagnosis.

### Pathological findings

**Fig. 1:**
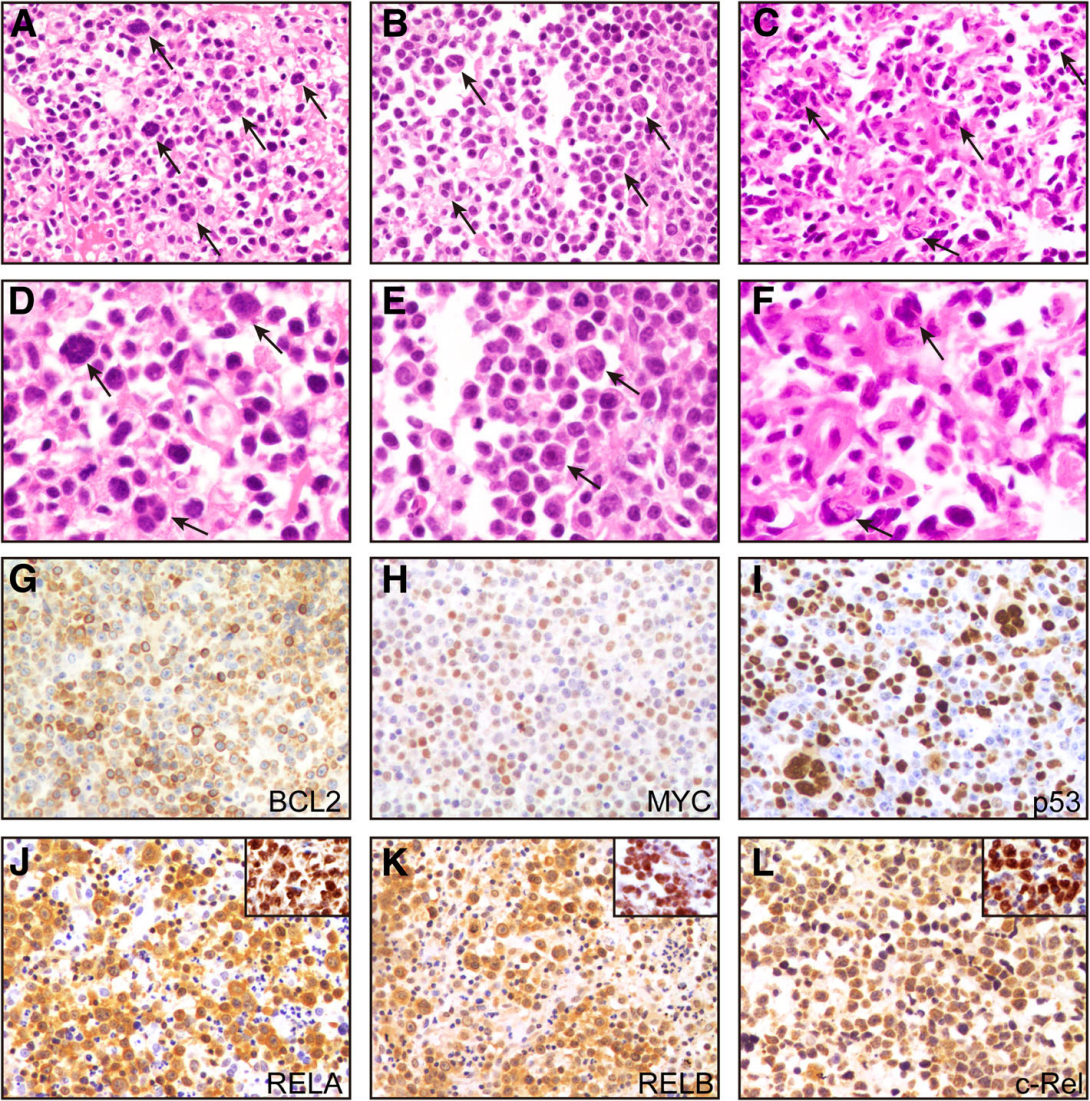
Representative hematoxylin and eosin (H&E) and immunostaining analysis of primary CNS A-DLBCL. (**a**-**c**) All three cases showed scattered binucleated and multinucleated, bizarre Reed–Sternberg-like tumor cells (black arrow) in the background of ordinary DLBCL (**a**: patient 1, **b**: patient 2, **c**: patient 3). (**d**-**f**) A high power view of the anaplastic tumor cells of each case (**d**: patient 1, **e**: patient 2, **f**: patient 3). Tumor cells of patient 3 showed a diffuse strong positivity for BCL2 (**g**), MYC (**h**) and p53 (**i**). Patient 2 showed positive staining for the NF-κB subunits RELA (**j**), RELB (**k**) and c-Rel (**l**) in both cytoplasm and nucleus. A positive control of each immunostaining for anaplastic DLBCL tissues inset at the upper right corner

All 3 patients showed diffuse infiltration in a sheet-like pattern and replacement of the brain parenchyma by large atypical lymphoid cells. At the edge of the tumor or in areas with a low density of tumor cells, characteristic features, including tumor cell infiltration in perivascular spaces, were observed. Polymorphic large polygonal tumor cells with anaplastic features were observed and scattered in the background of ordinary DLBCLs in all 3 patients, as described by Li et al. [6]. These cells were characterized by single or multiple irregular giant nuclei with hyperchromatin, thick nuclear membranes, and large nucleoli, which were similar to Reed-Sternberg-like cells (Fig. 1a-f).

The immunohistochemistry results are listed in Table 1. All 3 patients were strongly positive for CD20 and negative for CD3, ALK and EBER. According to Hans [11] and Choi algorithms [12], patients 2 and 3 demonstrated a non-GCB immunophenotype, whereas patient 1 demonstrated a GCB immunophenotype. The Ki-67 proliferation rate was generally high, ranging from 70 to 90%. The CD30 expression was positive in patient 1 but negative in patients 2 and 3. All 3 patients tested positive for both BCL2 (Fig. 1g) and MYC (Fig. 1h) (double-expressor). With respect to p53 staining, patient 1 had completely negative staining, patient 2 had variable expression (> 50%), and patient 3 had diffusely positive staining (Fig. 1i). Of the three NF-κB subunits including RELA, RELB and c-Rel, all 3 patients expressed at least two subunits of NF-κB in both cytoplasm and nucleus (patient 3 was negative for c-Rel) (Fig. 1j-l), indicating the nuclear translocation and activation of the NF-κB pathway.

**Table 1:** Morphologic, Immunophenotypic, and Molecular Genetic Characteristics of 3 Patients With Primary CNS A-DLBCL

	Cell of Origin		Immunohistochemistry									FISH			Mutation Statuses				
Patient	Hans	Choi	CD5	CD30	Ki-67	BCL2	MYC	P53	RELA	RELB	c-Rel	*MYC*	*BCL2*	*BCL6*	*TP53*	*MYD88*	*CD79B*	*CARD11*	*EZH2*
															(exon 5–10)	(exon 5)	(exon 5)	(exon 5–9)	(exon 16)
1	GCB	GCB	–	+	90%	+	+	All negative	+	+	+	extracopy	extracopy	extracopy	WT	L265P	WT	WT	WT
2	Non-GCB	Non-GCB	–	–	80%	+	+	Variable>50%	+	+	+	extracopy	extracopy	extracopy	WT	L265P	WT	WT	WT
3	Non-GCB	Non-GCB	–	–	70%	+	+	Diffuse	+	+	–	extracopy	no	split	R273C	L265P	WT	WT	WT

Abbreviations: *GCB* germinal center B cell, *WT* wild type

**Fig. 2:**
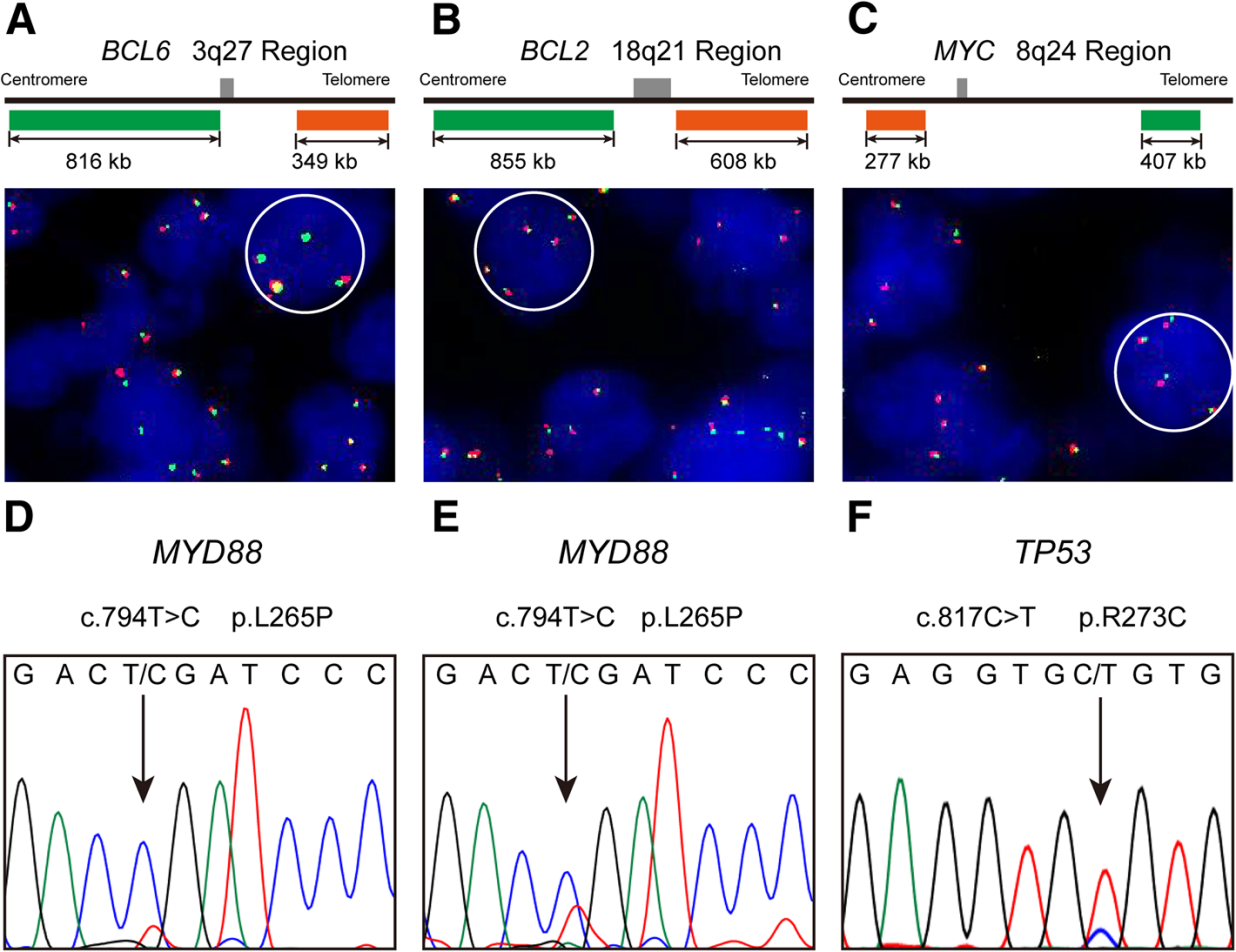
Representative results of fluorescence in situ hybridization (FISH) analysis and Sanger sequencing of primary CNS A-DLBCL. The FISH analysis for patient 1 showed extra copy signals for *BCL6* (**a**), *BCL2* (**b**) and *MYC* (**c**). *MYD88* L265P mutations in patients 1 (**d**) and 2 (**e**), in which a CTG (leucine) codon was changed to a CCG (proline) codon. *TP53* R273C mutation of patient 3 (**f**), in which a CGT (arginine) codon was changed to a TGT (cysteine) codon

The results of FISH and mutation status are listed in Table 1. Patients 1 and 2 had extra copies for all *MYC*, *BCL2* and *BCL6* genes (Fig. 2a-c), Patient 3 had an extra copy of *MYC* and a translocation of *BCL6*. With respect to mutation status, all 3 patients were successfully amplified for *TP53* exons 5–10, *MYD88* exon 5, *CD79B* exon 5, *CARD11* exons 5–9 and *EZH2* exon 16. *MYD88* L265P mutations were detected in all 3 patients, and patient 3 displayed the *TP53* R273C mutation (Fig. 2d-f). No mutations were identified for *CD79B*, *CARD11* and *EZH2* in these patients.

## Discussion

Primary CNS DLBCL is a rare entity with poor prognosis and needs a better understanding of the genetic characteristics and prognostic markers [13]. In terms of morphology, primary CNS A-DLBCL is an extremely uncommon lymphoma that has been described in only two other case reports to date but without intact clinicopathologic characteristics [7, 8]. In the 3 cases of this study, the characteristic features of perivascular space infiltration with bizarre-shaped tumor cells led to the diagnosis of primary CNS A-DLBCL after excluding systemic DLBCL with CNS involvement. It is important to realize the existence of primary CNS A-DLBCL to avoid being misdiagnosed as ALCL or undifferentiated carcinoma. The former is positive for CD30 and/or ALK and negative for B-cell markers, whereas primary CNS A-DLBCL expresses B-cell markers with negativity for ALK. Negativity for cytokeratin is helpful in distinguishing this type of DLBCL from undifferentiated carcinoma [14].

In our previous study of 35 patients with A-DLBCL, we defined A-DLBCL based on morphology and recognized the distinctiveness of this neoplasm from ordinary DLBCLs in terms of genetic alterations and biologic features, which contained a high incidence of p53 positivity and MYC/BCL2 double-expressor and a high frequency of *TP53* mutations and concurrent *MYC* and *BCL2* and/or *BCL6* abnormalities [6]. In the 3 patients with primary CNS A-DLBCL in this study, the concurrent of *MYC* and *BCL2* and/or *BCL6* abnormalities and MYC/BCL2 double-expressor occurred in all 3 patients, in which two patients had *MYC*/*BCL2*/*BCL6* triple extra copies, and one patient had *MYC* extra copy and *BCL6* translocation. The pattern of genetic abnormalities of these genes was overwhelmingly having an extra copy except *BCL6* translocation in case 3, which is consistent with translocations recurrently affecting *BCL6* and *IG* genes, whereas *MYC* translocations are rare and *BCL2* translocations are absent in primary CNS DLBCL [15–17]. The prognostic value of gain/amplification of *MYC* is still unclear in DLBCL, and some studies reported that gain/amplification of *MYC* is not associated with a poor prognosis [18–20]. Moreover, *BCL2* gain/amplification is associated with cell-of-origin–specific (activated B-cell-like subtype) clinical effect in R-CHOP-treated DLBCL [21]. In our previous study about A-DLBCL [6], *MYC/BCL2/BCL6* triple extra copies were found in 3 cases, and the overall survival is 5, 9, and 11 months, similar to the very aggressive disease course and poor prognosis of primary CNS A-DLBCL cases in this study, suggesting that in this specific morphology (anaplastic variant), *MYC/BCL2/BCL6* copy number alterations may be an adverse prognostic factor, but more cases are needed to confirm this finding.

We detected one patient with diffuse positive p53 immunostaining and *TP53* gene mutation (R273C). The *TP53* mutation rate is approximately 20% in DLBCL [22] and approximately 26.7–37.2% [23, 24] in primary CNS DLBCL. However, few studies that report *TP53* mutation status in PCNSL reveal incidences < 10% [25, 26]. Moreover, we also found one patient had a *TP53* null phenotype (completely negative), similar to that seen in high grade serous carcinomas [27]. Tumors that were completely negative for p53 IHC expression had a mutation of *TP53* in 65% of cases and wild-type *TP53* in 11%. Therefore, complete absence of p53 immunostaining is commonly associated with a *TP53* mutation status. However, the *TP53* exon 5–10 of this patient was wild type by sanger sequencing, suggesting that there may be a wild-type *TP53* or variations in other positions besides exon 5–10*. TP53* mutations alter the wild-type p53 protein structure and disrupt its function, which is implicated in lymphomagenesis and disease progression [28]. The genomic instability driven by the *TP53* mutation and *MYC*, *BCL2*, and *BCL6* abnormalities might partially explain the poor clinical course and may be good prognostic factors in patients with primary CNS A-DLBCL. It seems that these genetic alterations probably occurred more frequently in primary CNS A-DLBCL patients than in patients with ordinary primary CNS DLBCL without anaplastic features, suggesting that the morphology is closely related to genetic alterations and disease prognosis. However, as there are rare reports of primary CNS A-DLBCL, it is necessary to confirm our observations with a large number of cases in the future.

In primary CNS DLBCL, mutations of *MYD88* are believed to promote lymphomagenesis via constitutive activation of the NF-κB pathway. MYD88 is an adaptor molecule in the Toll-like receptor pathway that mediates interleukin-1 receptor signaling [29]. A somatic mutation of *MYD88* is found in 67–86% [26] [30, 31] of primary CNS DLBCL, and the most common mutation site is L265P. In this study, all 3 primary CNS A-DLBCL patients displayed mutations in *MYD88* L265P, which is consistent with the high frequency of *MYD88* alterations reported in the literature for primary CNS DLBCL. The high frequency of *MYD88* mutations in primary CNS A-DLBCL are probably not unique to the anaplastic variant, but mainly due to the site of CNS. Because the frequency of *MYD88* mutation appeared to be higher in this small series than that of A-DLBCL in our previous study (100% vs 20%) [6]. The activation of the NF-κB pathway leads to the nuclear translocation of NF-κB dimers and subsequently initiates activation of other target genes [32]. In all 3 cases in this study, tumor cells expressed at least 2 nuclear NF-κB subunits, suggesting that NF-κB signaling activation is an almost universal feature of primary CNS A-DLBCL. Given the high prevalence of *MYD88* mutations in primary CNS A-DLBCL, treatment with ibrutinib, which inhibits Bruton’s Tyrosine Kinase (BTK) and further suppresses NF-κB and STAT3 activation and tumor growth, or treatment with bortezomib, which is effective for *CD79B*/*MYD88*^L265P^ double-mutant DLBCLs [33], could be considered in patients with primary CNS A-DLBCL [34].

Current treatment for primary CNS DLBCL includes surgery followed by chemotherapy with methotrexate-based regimens, with or without adjuvant radiation therapy [3, 13]. Unlike other brain tumors, primary CNS DLBCL regularly has a good response to chemotherapy and radiation therapy, but in comparison with lymphomas outside the CNS, survival is not as prominent, partly due to inadequate penetration of the blood-brain barrier [35]. Most protocols report a median progression-free survival of approximately 12 months and an overall survival (OS) of approximately 3 years. However, the prognosis for primary CNS DLBCL that has failed first-line therapy remains poor, with a low median OS of 12 months [13]. In the 3 patients with primary CNS A-DLBCL in this study, the median OS was only 5 months, which is much lower than that of primary CNS DLBCL and A-DLBCL (16 months) [6]. The International Prognostic Index (IPI) score used for determining the prognosis of patients with aggressive NHL seems not fit into primary CNS DLBCL. The IELSG score, including five parameters (age, ECOG performance status, LDH level, CSF protein concentration, deep brain involvement), is widely used to predict outcomes and to better stratify patients. The presence of 0 to 1, 2 to 3, or 4 to 5 adverse risk factors correlates with 2-year survival rates of 80, 48%, or 15%, respectively [9]. In our 3 cases, the scores of patients were all 4, suggesting an aggressive disease course and poor survival, in accordance with intricate genetic alterations and adverse prognosis factors.

## Conclusions

In summary, patients with primary CNS A-DLBCL may often have a MYC/BCL2 double-expressor and concurrent *MYC* and *BCL2* and/or *BCL6* genetic abnormalities, as well as constitutive activation of the NF-κB pathway. The importance of this type of lymphoma is likely underestimated, and recognition is likely important because most patients follow a very aggressive disease course and have a poor prognosis. In the future, a large number of cases should be analyzed, and an evaluation of molecular genetic alterations could help with practical and therapeutic implications for primary CNS A-DLBCL.

## Abbreviations

A-DLBCL: Anaplastic variant of DLBCL
ALCL: Anaplastic large cell lymphoma
BTK: Bruton’s Tyrosine Kinase
CNS: Central nervous system
CSF: Cerebrospinal fluid
DHL: Double-hit lymphoma
DLBCL: Diffuse large B-cell lymphoma
ECOG: Eastern Cooperative Oncology Group
HD-MTX: High-dose methotrexate
IELSG: International Extranodal Lymphoma Study Group
IPI: International Prognostic Index
LDH: Lactate dehydrogenase
NHL: Non-Hodgkin lymphoma
OS: Overall survival
PD: Progressive disease
PR: Partial remission
THL: Triple-hit lymphoma
WBRT: Whole-brain radiotherapy
